# Intensity Demodulated Refractive Index Sensor Based on Front-Tapered Single-Mode-Multimode-Single-Mode Fiber Structure

**DOI:** 10.3390/s18072396

**Published:** 2018-07-23

**Authors:** Jing Kang, Jiuru Yang, Xudong Zhang, Chunyu Liu, Lu Wang

**Affiliations:** 1 College of Electronics Engineering, Heilongjiang University, Harbin 150080, China; 2171241@s.hlju.edu.cn (J.K.); 2161304@s.hlju.edu.cn (X.Z.); 2004061@hlju.edu.cn (C.L.); 2 Key lab of Electronics Engineering, College of Heilongjiang Province, Heilongjiang University, Harbin 150080, China

**Keywords:** fiber refractive index sensor, modal interference, intensity demodulation, SMS, taper

## Abstract

A novel intensity demodulated refractive index (RI) sensor is theoretically and experimentally demonstrated based on the front-tapered single-mode-multimode-single-mode (FT-SMS) fiber structure. The front taper is fabricated in a section of multimode fiber by flame-heated drawing technique. The intensity feature in the taper area is analyzed through the beam propagation method and the comprehensive tests are then conducted in terms of RI and temperature. The experimental results show that, in FT-SMS, the relative sensitivity is −342.815 dB/RIU in the range of 1.33~1.37. The corresponding resolution reaches 2.92 × 10^−5^ RIU, which is more than four times higher than that in wavelength demodulation. The temperature sensitivity is 0.307 dB/°C and the measurement error from cross-sensitivity is less than 2 × 10^−4^. In addition, fabricated RI sensor presents high stability in terms of wavelength (±0.045 nm) and intensity (±0.386 dB) within 2 h of continuous operation.

## 1. Introduction

Fiber refractive index (RI) sensors play a significant role in the fields of biology, chemistry, and medicine [1]. And the schemes based on fiber Bragg gratings [2], long-period fiber gratings [3], photonic crystal fiber [4,5], multimode interference (MMI) [6] and surface Plasmon resonance (SPR) [7,8] are frequently reported. Comparatively, the MMI-based sensors have received great attention due to the advantages of high sensitivity, low cost, and ease of fabrication. A typical MMI sensor can be formed through splicing a section of multimode fiber (MMF) with two pieces of single mode fiber (SMF), namely single-mode-multimode-single-mode (SMS) fiber structure [9,10,11]. Multipath interference then occurs among high-order core modes and brings high sensitivity to the ambient parameters.

To be applied in RI sensing, Shao cascades thin-core fiber with SMS fiber structure and forms the composite modal interference [12]. Wang and Yang comprehensively analyzed and compared the RI sensitivity of tapered-SMS fiber structures [9,13,14,15]. Moreover, the tapered-multi-core and multi-taper-based schemes have been respectively proposed, and the over-200-nm/RIU sensitivity was obtained in References [16,17]. Recently, the flame-heated drawing technique was adopted to further enhance the sensitivity of MMI based sensors. Fu reported a U-shape fiber humidity sensor with waist-diameter of 4.75 μm [18]. Zhang used a tapered polarization maintaining fiber to measure the concentration of ammonia [19]. In addition, the fiber structures with higher strain and curvature sensitivities are presented in [20,21].

In this paper, an intensity demodulated RI sensor is proposed to gain higher measured precision based on the front-taper SMS (FT-SMS) fiber structure, in which a taper is fabricated in the front of a section of MMF by flame-heated drawing. The composite modal interference is formed, and the intensity feature in taper area is then analyzed through the beam propagation method. The experimental results show that, in FT-SMS, the intensity of fringe is dramatically decreased with the increased external RI, and the relative sensitivity reaches −342.815 dB/RIU in the range of 1.33–1.37. Compared to wavelength demodulation, four times enhancement in detecting resolution is obtained. In addition, the measured error from cross-sensitivity is limited within 2 × 10^−4^, owing to low-temperature sensitivity. This fabricated RI sensor, also, presents high stability in terms of wavelength (±0.045 nm) and intensity (±0.386 dB).

## 2. Principles

**Figure 1:**
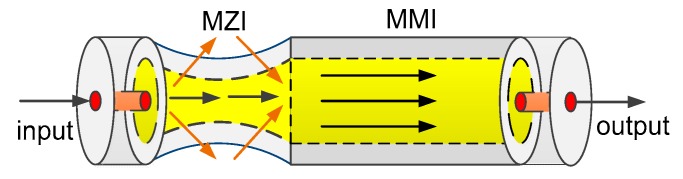
Scheme diagram of the front-tapered single-mode-multimode-single-mode (FT-SMS) fiber structure.

The FT-SMS fiber structure is illustrated in Figure 1. A taper is located at the front end of MMF, which includes two transition areas and a taper-waist area. Based on the theory of evanescent wave filed, in the first transition area the inputted light will partly leak out and excite high-order cladding modes. When transmitting in the second transition area, the cladding modes can re-couple into the fiber core [19]. Then a Mach-Zehnder interferometer (MZI) is formed in the taper area, and both the intensity and wavelength will be sensitive to the change of external RI.

According to Reference [22], the light intensity of MZI can be expressed as:

(1) $$I=I_{1}+I_{2}+2\sqrt{I_{1}+I_{2}}\cos\Delta \varphi$$

where *I*_1_ and *I*_2_ represent the intensity of the core and cladding modes, respectively. Δφ is the phase difference between the core and cladding modes and can be written as:

(2) $$\Delta \varphi =\frac{2\pi \Delta n_{eff}L_{t}}{\lambda }$$

where *λ* is the wavelength of incident light, $\Delta n_{eff}\;$ is the difference of effective RI between the core and cladding modes and $L_{t}$ is the length of taper area. Therefore, the corresponding interfered wavelength will be:

(3) $$\lambda_{MZI}=\frac{2\Delta n_{eff}L_{t}}{2j+1}$$

where *j* is the order of cladding mode. Moreover, an MMI will occur in the residual MMF due to the multipath difference among high-order core modes [21]. Here, the diameter and length of taper-waist are denoted by $D_{w}$ and $\;L_{w}$, respectively. The lengths of transitional areas are Z and the initial length of MMF is L. From Reference [23], the interfered wavelength between the $m_{th}$ and $n_{th}$ high-order core modes will be:

(4) $$\lambda_{MMI}=\frac{8\left(2N+1\right)n_{co}r^{2}}{\left(m-n\right)\left[2\left(m+n\right)-1\right]\left(L-L_{t}\right)}\;(m>n)$$

where N is an integer, $n_{co}$ and *r* are the effective RI and radius of fiber core, respectively. Then the wavelength spacing of MMI can be written as:

(5) $$\Delta \lambda_{MMI}=\frac{16n_{co}r^{2}}{\left(m-n\right)\left[2\left(m+n\right)-1\right]\left(L-L_{t}\right)}\;(m>n)$$

**Figure 2:**
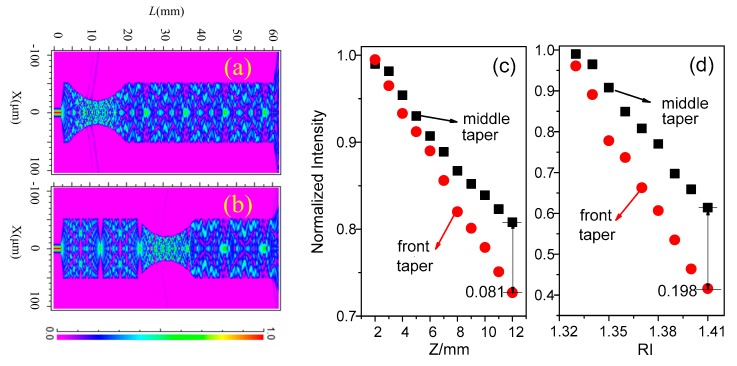
Interference patterns in (**a**) front and (**b**) middle tapers, and the normalized intensity of tapered fiber structures with varied (**c**) transitional area length and (**d**) external refractive index (RI).

Further, assume that the length of MMF is 50 mm with the diameter of 105/125 μm, $n_{co}=1.4662$ and $n_{cl}=1.4450$. The $n_{co}$ of SMF is 1.4502 with the diameter of 8.3/125 μm. Then by using the beam propagation method (the incident wavelength is 1550 nm, and the computational rectangular area is 0.105 × 61.798 mm^2^ with the mesh area of 0.2 μm^2^), the intensity features of SMS with front and middle tapers are compared under the varied $L_{t}\left(=L_{w}+Z\right)$ and external RI. Figure 2a,b shows the interference patterns in the front and middle tapers, and it is clear that there is more energy leaked in the front-tapered (FT) structure. We then set $L_{w}$ = 8 mm, the radius of taper waist can be calculated by Equation (6), which is decreased with the increase of Z [13].

(6) $$R\left(Z\right)=R_{0}e^{\left(\frac{-Z}{L_{w}}\right)}$$

where *R*_0_ is the radius of MMF. The changes in normalized intensity are shown in Figure 2c with the varied Z (from 2 to 12 mm). We observe that the intensities are decreased in both tapered SMS structures with the rise of Z, but the difference of them is continuously increased (the maximum 0.081 occurs at *Z* = 12 mm). We further set Z=6.5 mm and the similar results are presented in Figure 2d with the varied external RI (from 1.33 to 1.41). The intensity deduction in FT structure is clearly larger than that in middle taper and the maximum difference reaches 0.198 when RI = 1.41, which means that a higher RI sensitivity may be gained in FT-SMS.

## 3. Fabrication

**Figure 3:**
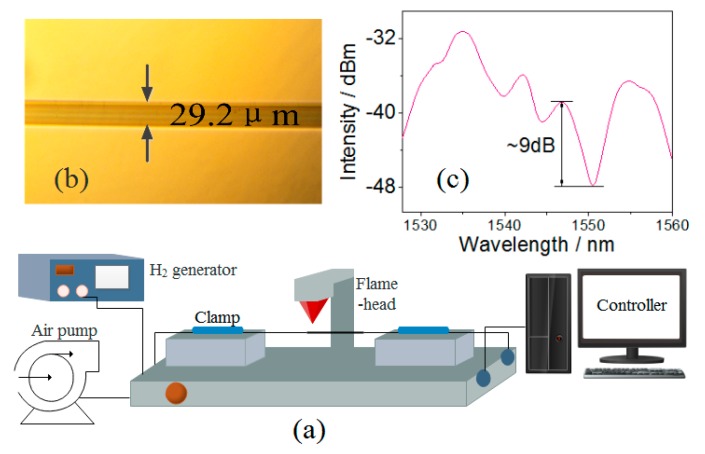
(**a**) Schematic diagram of fabrication of FT-SMS; (**b**) the CCD image of taper waist; (**c**) the transmission spectrum of FT-SMS.

A 50-mm un-coated MMF (MM-S105/125-12A, Nufern, Hartford, CT, USA) was first spliced with two pieces of SMF (SMF-28, Corning, New York, NY, USA) by using a commercial fusion splicer (KL-280, Geelong, Nanjing, China). This SMS fiber structure was then placed into a melt–drawing machine (KF-FBT). As shown in Figure 3a, the front end of MMF is positioned under the center of flame-head. We set the speeds of drawing and hydrogen flow to 300 μm/s and 150 mL/min, respectively. The SMS fiber was then evenly stretched, and the controller showed the stretching length was 7.8 mm and $L_{t}$ = 17.8 mm, accordingly $L-L_{t}$ = 3.22 cm. Figure 3b is the CCD (Coupled charge device) image of the fabricated taper and its waist-diameter is 29.2 μm. After 5-h annealing, the transmission spectrum of FT-SMS (in air) was tested and shown in Figure 3c. It is obvious that there is a main interference fringe located at 1550.51 nm with the contrast ratio of ~9 dB.

## 4. Experiments and Results

**Figure 4:**
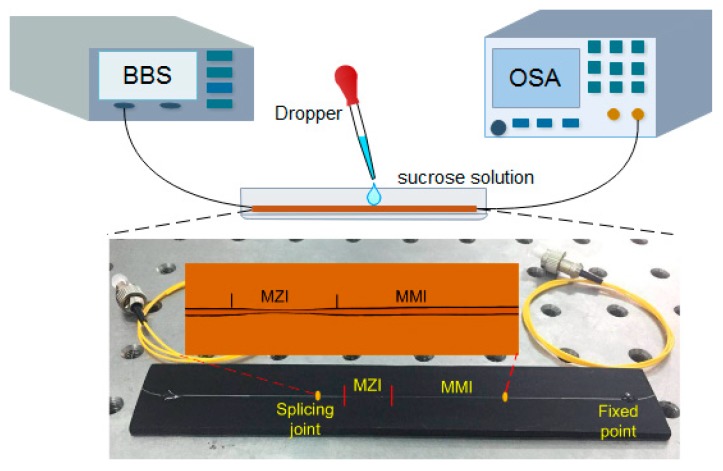
Experimental setup for RI measurement.

As shown in Figure 4, the sensing head was flatly placed and fixed onto a glass slide by epoxy rean sin adhesive. The broadband source (BBS, homemade, operated in 1520–1565 nm) was fixed at 50 mA, and the room temperature was kept at 23 ± 0.2 °C. The RI test was then performed through varying the concentration of sucrose solution from 0 to 20% (the corresponding RI is 1.33–1.37). The shifts of interference fringes were recorded by an optical spectrum analyzer (OSA, Agilent 86142B, with the resolution of 0.06 nm/0.01 dB).

**Figure 5:**
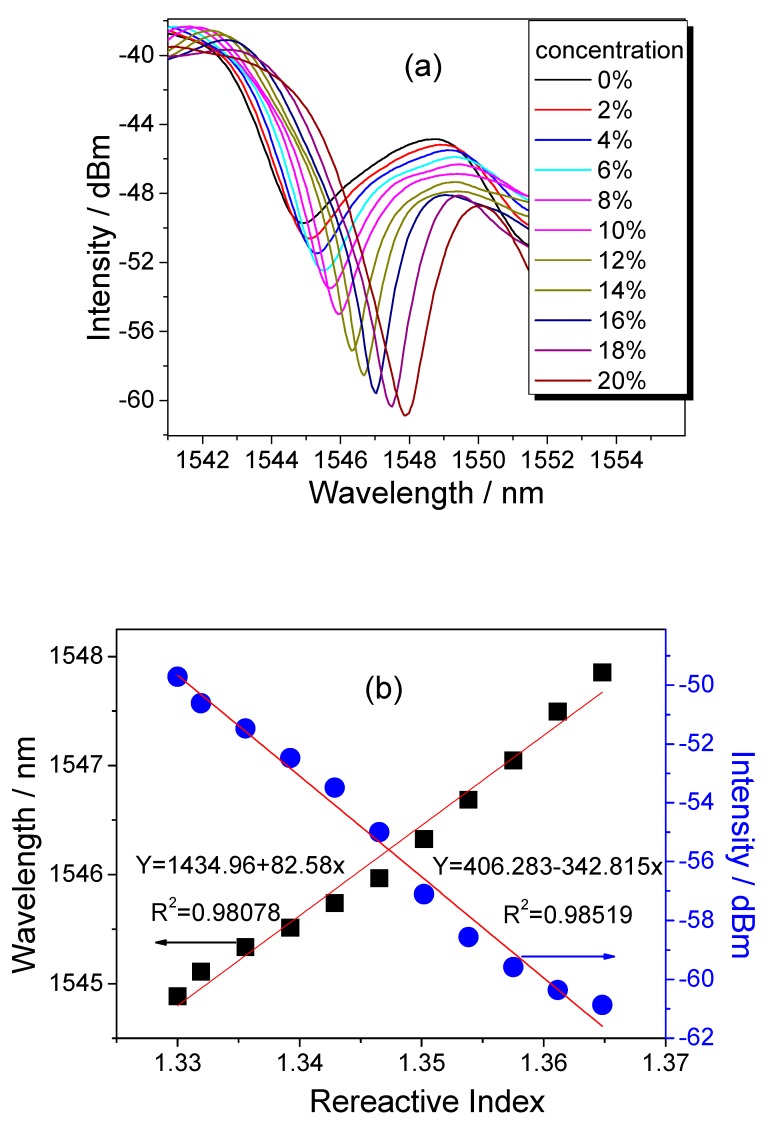
(**a**) Shift of interference fringe with different concentration and (**b**) the sensitivity and linearity of wavelength and intensity with varied RI.

In Figure 5a, the dip of interference fringe moves toward long wavelength with the increase of solution concentration, but the intensity of fringe is quickly decreased. The total deduction reaches 11.15 dB (from −49.72 to −60.87 dB). According to Figure 5b, the sensitivity is −342.815 dB/RIU, and the linearity is 0.985. Because of the resolution of OSA (0.01 nm), the detection limit reaches 2.92 × 10^−5^ RIU. Comparatively, the dip shifts ~2.97 nm in the range of 1.33–1.37. By calculation, the wavelength sensitivity is 82.58 nm/RIU with the linearity of 0.981. And the corresponding resolution is 7.27 × 10^−4^ RIU. These results mean that the detection limit is enhanced about four times when the intensity demodulation is adopted.

**Figure 6:**
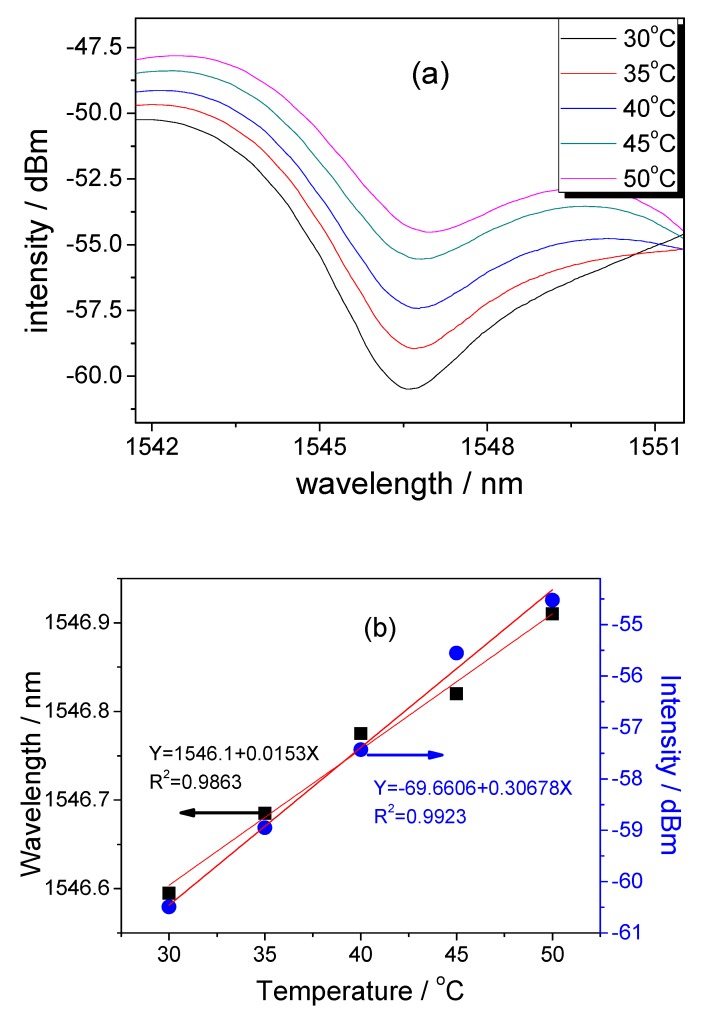
(**a**) Shift of interfered fringe with varied temperature and (**b**) the corresponding sensitivity and linearity of wavelength and intensity.

Further, the FT-SMS based sensor was placed onto a heater (DFD-7000, LICHEN, Shanghai, China) and its temperature feature was characterized and demonstrated. From Figure 6a, as the temperature increases, the wavelength shifts toward long wavelength and the intensity also increases ~5.97 dB due to the expansion of fiber core. Figure 6b shows the corresponding sensitivity is 0.307 dB/°C with the linearity of 0.992. The wavelength shift of dip is 0.315 nm in the range of 30–50 °C and the sensitivity is 15.3 pm/°C with the linearity of 0.986.

Considering the ambient drift of ±0.2 °C, the intensity fluctuation will be 0.061 dB/RIU from cross-sensitivity, and the measurement error is limited in 0.175‰ in our sensor. Further, from Reference [24], the variations of temperature (ΔT) and external RI (Δn) can be simultaneously measured by the inversion matrix:

(7) $$\left[\begin{array}{c} \Delta T\\ \Delta n \end{array}\right]=\frac{1}{D}\left[\begin{array}{cc} k_{In} & -k_{\lambda n}\\ -k_{IT} & k_{\lambda T} \end{array}\right]\left[\begin{array}{c} \Delta \lambda \\ \Delta I \end{array}\right]$$

where Δλ is wavelength shift, ΔI is intensity change. $D=k_{\lambda T}k_{In}-k_{IT}k_{\lambda n}$, where *k_λT_* = 0.015, *k_λn_* = 82.58 are the wavelength sensitivities in temperature and RI, and *k_IT_* = 0.307, *k_In_* = −342.82 are the intensity sensitivities in temperature and RI. Therefore, Equation (7) is changed as:

(8) $$\left[\begin{array}{c} \Delta T\\ \Delta n \end{array}\right]=\frac{1}{-30.49}\left[\begin{array}{cc} -342.82 & -82.58\\ -0.307 & 0.015 \end{array}\right]\left[\begin{array}{c} \Delta \lambda \\ \Delta I \end{array}\right]$$

**Figure 7:**
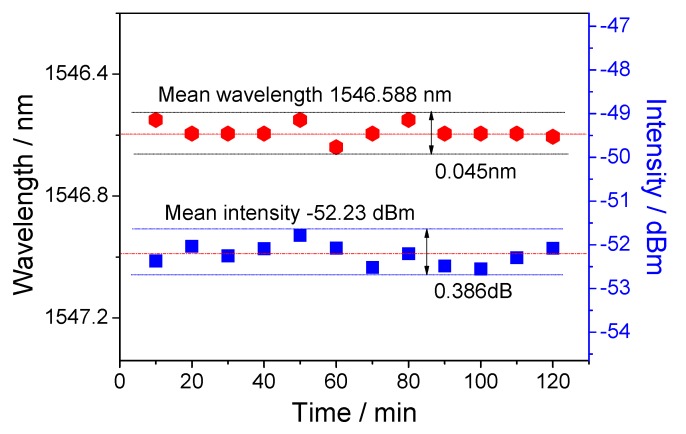
Stabilities in terms of wavelength and intensity within 2 h.

Table 1 compares the numerical results of several tapered fiber structures, and it is obvious that our sensor based on intensity demodulation presents a higher mean sensitivity and detecting resolution in the range of 1.33–1.37. Finally, considering the influence of light source power fluctuation and wavelength drift under a long working time [25], a 120-min stability test was performed at room temperature, and the numerical results are shown in Figure 7. By calculation, the fluctuations of wavelength and intensity are ±0.045 nm and ±0.386 dB, respectively. It is worth noting that the sensor head can be packaged in a capillary to enhance its durability [26].

## 5. Conclusions

In this paper, a novel RI sensor is fabricated based on FT-SMS fiber structure through flame-heated drawing technique. Because of the effect of evanescent wave filed, this RI sensor presents ultrahigh sensitivity and linearity in the range of 1.33–1.37. And the detecting resolution reaches 2.92 × 10^−5^ RIU, which is more than four times higher than that in wavelength demodulation. Moreover, small temperature sensitivity (0.307 dB/°C) and high stability (±0.045 nm/±0.386 dB) are simultaneously demonstrated in FT-SMS fiber structure. Such low cross-sensitivity and instability indicate that the fabricated sensor is a promising and practical device for the applications of biochemical sensing and environmental monitoring.

## Figures and Tables

**Table 1:** Comparisons of sensitivity and resolution.

Structures	Detection RI Range	Sensitivity (nm/RIU)	Sensitivity (dB/RIU)	Resolution (RIU)	Refs.
tapered fiber with localized SPR	1.333–1.403	51	—	3.2 × 10^−5^	[7]
SMS by chemical etching	1.33–1.432	—	182.48	5.48 × 10^−5^	[9]
SMS cascaded TCF	1.333–1.403	148.27	112.37	1.34 × 10^−4^	[12]
middle-tapered SMS	1.33–1.44	487	—	2.05 × 10^−5^	[13]
	~1.44	1913		5.23 × 10^−6^	
tapered multi-core fiber	1.345–1.377	171.2	63.59	2.92 × 10^−4^	[16]
multi-tapered SMS	1.333–1.375	261.9	170.2	—	[17]
tapered small-core fiber	1.34–1.346	1198.3	—	0.83 × 10^−5^	[27]
	1.375–1.384	2123.6	—	4.7 × 10^−6^	
	1.43–1.432	19212.5	—	5.2 × 10^−7^	
tapered fiber with SPR	1.33–1.391	2238.4	—	—	[28]
	1.386–1.416	866.1	—	—	
front taper-SMS	1.33–1.37	82.58	−342.8	2.92 × 10^−5^	Our work
